# Options for sustaining solar-powered mosquito trapping systems on Rusinga Island, Western Kenya: a social dilemma analysis

**DOI:** 10.1186/s12889-018-5218-y

**Published:** 2018-03-06

**Authors:** Prisca A. Oria, Michiel Wijnands, Jane Alaii, Cees Leeuwis

**Affiliations:** 1 Knowledge, Technology and Innovation Group, Wageningen University and Research, Wageningen, The Netherlands; 2 Context Factor Solutions, Nairobi, Kenya

**Keywords:** Sustainability, Solar, Mosquito, Traps, Malaria, Social dilemma, Community, Kenya

## Abstract

**Background:**

In 2012, a donor-supported proof of principle study was launched to eliminate malaria from Rusinga Island, western Kenya, using solar-powered mosquito trapping systems (SMoTS). SMoTS, which also provided power for room lighting and charging mobile telephones, were installed in houses. In view of the involvement of individual and collective benefits, as well as individual and collective maintenance solutions, this study qualitatively examined preferences of some project stakeholders towards SMoTS sustainability components to see if and how they related to social dilemma factors.

**Methods:**

The data were collected through participant observation, semi-structured interviews and focus group discussions.

**Results:**

The results show that respondents largely preferred individual solutions to various aspects of maintenance. Selective collective solutions such as table banking groups were considered positively for mobilising financial resources for maintenance, but respondents were hardly willing to contribute financially to a collective entity. Few people saw a meaningful role for a collective governing body; people preferred to rely on individual household responsibility and private service delivery for repairs and stocking spare parts. An overriding concern was that people lacked trust in other community members, leaders and/or technicians who would be employed by a governing body. Respondents also had little confidence that a governing body or saving group could effectively impose sanctions to misappropriation of funds, poor leadership, defecting group members or technicians that might abuse a salaried position.

**Conclusion:**

There seemed to be linkages between preferences towards organising various components of SMoTS sustainability and known hindrances to addressing social dilemmas. This posed considerable challenges to organising the sustainability of this innovative malaria control strategy.

**Trial registration:**

NTR3496.

## Background

In 2012, a four-year proof of principle study was launched to eliminate malaria from Rusinga Island, using solar-powered mosquito trapping systems (SMoTS) [1, 2]. SMoTS were installed in houses to also provide power for room lighting and charging mobile telephones [3]. The project was launched with core support from donor funding and the research phase ended in December 2015, after which project funding for SMoTS parts and repairs ceased. A project-initiated community advisory board (CAB) [4] spearheaded community development of a sustainability plan.

Demonstration programmes are usually deployed in real life conditions but with immense technical and financial resource support to ensure optimal conditions to enable learning on the side about long term feasibility. However, when sustainability is not assessed, it remains questionable whether community efforts can be sustained long enough to have a lasting effect [5]. While not all research projects need to be sustained, the current project was unique in that although its main aim was malaria elimination, it provided an unrelated benefit of electricity for house lighting and telephone charging. While these benefits were mainly at the level of individual homestead, the benefits of malaria control accrued to the wider public.

Substantial time and resources are often invested in programme development, adaptation and implementation. It is important, as an integral part of the programme planning process, to identify factors that promote the integration and maintenance of new programmes into community setting [6–8].

### Social dilemma theory: public goods dilemma

Social dilemmas are situations in which the rational pursuit of self-interest can lead to collective disaster [9]. A public good can be provided only if group members contribute something towards its provision; however, both contributors and non-contributors may benefit [10]. Unlike the SMoTS electrification which is private and enjoyed exclusively at the individual household level, malaria control is a public benefit realised beyond the installed house. Each installed household ensures that the mosquito trap is always in optimum working condition to assure adequate coverage of traps in the area.

While in a classic public goods dilemma maximum benefits are accrued through collective organisation, SMoTS had distinguishing characteristics; (a) some sustainability components could be organised individually and others collectively, (b) provided double incentives of malaria control and electrification, and (c) presented an immediate (electrification) and a long-term (malaria control) incentive. Critical sustainability components for SMoTS included: ownership, repair of installed SMoTS, stocking and selling parts, and financing SMoTS repairs and spare parts. Each of these factors could be arranged individually and/or collectively.

A number of factors influence whether people choose short-term individual benefit or longer term collective benefit [11]. Factors that facilitate group cooperation in social dilemmas include; trust in partners, smaller group size, enhanced in-group identity, effective leadership, and possibility of sanctioning. Strong perceptions of trust positively influence cooperation [11–15]. Cooperation decreases in large groups mainly because people feel less efficacious, are less identifiable and may feel negligible responsibility to pursue the group’s welfare [16–18]. Those with strong group identification have been found to invest more in public goods [15, 19], because their trust in others to do the same is high. Sanctioning and effective leadership are key factors in promoting cooperative behaviour [20–23].

This study qualitatively explored community aspirations for sustainability, and investigated whether the community preferred individual or cooperative solutions for organising sustainability components, and whether and how known social dilemma factors could be recognised in the reasoning of actors.

## Methods

The data reported here were collected as part of a multidisciplinary trial [1, 2] on which the social science studies assessed socio-cultural and behavioural aspects of design, adherence to use and maintenance of SMoTS.

### Study site and population

This study was carried out on Rusinga Island, western Kenya. The island covers an area of 44 km^2^. The project’s census estimated the population of the island at 23,337 individuals living in 4062 homesteads in May 2012 [24]. The trial targeted the whole island and each eligible homestead received a SMoTS. Roll-out of SMoTS begun in June 2013 and was completed in June 2015 after 4, 358 houses were installed [1].

Malaria is typically endemic in this region and transmission occurs throughout the year [25, 26]. The main economic activities are fishing, small-scale trade and traditional subsistence agriculture. While fishing is almost entirely practiced by men, women dominate fish trading. Dholuo is the main language spoken on the island. The study site has been described elsewhere [1, 3, 4, 24].

### Sampling and data collection

Questionnaires developed in English were translated into *Dholuo*. Corrections were made during fieldworker training and piloting. Participant observation, semi-structured interviews and focus group discussions (FGDs), in that sequence, were carried out. Each preceding process informed questions for the next. The data were collected in two phases. The first phase involved participant observation of routine CAB meetings to plan for the sustainability of SMoTS. A total of six meetings took place between August 2014 and June 2015.

The second phase which assessed preferences for sustainability options included 16 semi-structured interviews, comprising nine CAB members (6 males, 3 females) and seven community opinion leaders (4 males, 3 females). The CAB members purposively selected out of 16 were residents of the island and eligible to receive SMoTS. A variant of snow-ball sampling was used to select opinion leaders. After interviewing a CAB member, they were asked to provide names of community members perceived as opinion leaders. This list mostly resulted in names of males. Informal questioning of community members elicited names of female opinion leaders that were added to balance gender representation. Interviews lasted from 25 November through 8 December 2014. All interviews took place in a setting of the respondent’s choice, most often in their home. The questionnaire explored respondent’s understanding of sustainability of SMoTS, measures to improve prospects for sustainability, preferences towards organizing each sustainability component, and experiences with collective community initiatives.

Interview data were used to suggest four preliminary sustainability models presented to participants for evaluation during FGDs conducted from 8 through 15 January 2015. Community members explored potential advantages and disadvantages of each model. Six FGDs were conducted: four with residents of villages and two with residents of beaches (35 participants in total). The distinction between villages and beaches was made because an earlier study [3] showed a difference in perceptions and behaviours of residents of the two settings. Half of the FGDs in each location were with males. FGD participants had to be over 18 years old and have a SMoTS. Participants were randomly selected from the project SMoTS’ installation list.

Experienced bilingual research assistants conducted the interviews and moderated the group discussions in *Dholuo*.

### Data analysis

The research assistants transcribed verbatim narrative interviews directly from *Dholuo* into English, discussing any interpretation issues with the first two authors. To decrease investigator bias in the data analysis, we employed coding triangulation [27, 28]. Together PAO, MW and CL developed a thematic framework based on the interview topic guides which included relevant components of a framework for sustaining SMoTS (e.g. ownership arrangements, provision of spare parts, implementation of repairs, ensuring financial resource, etc.), possible ways of organising these sustainability components (through individual, collective or hybrid responsibilities) and likely factors informing stakeholder preferences. The latter derived from social dilemma theory, and included themes like trust, group size, leadership, possibility of sanctioning, etc. The resulting list was updated with new themes emerging from the data. Following the construction of the coding framework, PAO and MW independently analysed the transcripts of the CAB meeting notes, interviews and focus groups. Data analysis was conducted through manual coding and categorization of responses.

## Results

### Exploring views on sustaining SMoTS

Findings are presented according to the four sustainability components.

#### Ownership of SMoTS

Respondents portrayed preference that members of installed households should own SMoTS after the research phase ends because they were motivated to maintain the system and trained to care for them.

A CAB member said, *“It is not easy for someone not staying in an installed house to be assigned ownership as he may not be concerned and things may not proceed well. He may even take away the cables!”*

However, there were concerns that individual ownership may allow residents to migrate from Rusinga with SMoTS. SMoTS were deemed to be the property of Rusinga Island.

*“Some people received SMoTS even though they are not natives of Rusinga and if we allow individual ownership when the research ends, they will definitely leave with the system. These things should remain with us,”* said a CAB member.

Although collective ownership was deemed to offer a better environment for proper maintenance of SMoTS, the idea did not receive much prominence in discussions. Participants cited potential for non-cooperation by group members, but equally remarked that such challenges could be managed effectively through proper group regulations.

*“There should be a board that makes sure this thing (SMoTS) is sustained … not the beneficiary. The beneficiary should use it as a result of paying a small fee. Similar to Kenya Power where users pay a monthly fee to access electricity. I am a beneficiary but the project is not mine,”* said a CAB member.

*“As long as a person has a SMoTS installed in their house they must join a group. And as long as you are a member of a group, you must abide by the rules and regulations governing the group,”* said an opinion leader.

#### Repair of installed SMoTS

Respondents mainly indicated repairs should be done by project trained technicians or other local technicians, citing the importance of familiarity with the equipment.

*“Project technicians should be the only people to repair SMoTS. You are very likely to find an error if you use random experts to repair this equipment,”* said an opinion leader.

Three ways of paying technicians were proposed; per service by the household in need of repairs, monthly fee to a governing body, or through group savings. CAB members were reluctant to save in groups for repair costs mainly preferring to pay individually. Some opinion leaders, however, said they would save in groups for repairs.

*“The households will pay the technicians and it will be very cheap for each household because the technicians reside around here .... They are our sons and we know them,”* said a CAB member.

None of the interviewees thought it was a good idea to employ technicians full-time. Technicians would still make a living out of being paid per service because some were also fishermen and others businessmen.

*“We cannot employ them because we cannot sustain them. After they have done their job, we pay and finish with them,”* said an opinion leader.

#### Stocking and selling SMoTS’ parts

The majority of the interviewees said a shop in Mbita would be the most convenient place to obtain spare parts. The rest preferred the project to stock parts for sale to the community. While most respondents preferred a private shop, a few CAB members said this shop could be run by the CAB while others proposed a governing body. Households would pay a monthly fee to the CAB or governing body to purchase stock which community members would afterwards buy from the shop.

Respondents had no preference of the private person to run the shop although they suggested it would be better if the project approached a businessperson than community members who were perceived to have strong personal interests.

*“Because the community would talk nonsense ... the project should approach him because I will talk of my benefit, you will talk of yours but the project will approach him without a direct interest,”* said a CAB member.

#### Financing spare parts

To improve prospects for sustainability, in addition to paying technicians, money would also be needed to buy spare parts of SMoTS. There were three categories of responses in this category; through individual means, through group savings, and through donor support. Saving at the individual level was deemed most reliable but respondents pointed out that some members of the community such as the elderly and poor would require help to have sufficient money for spare parts, hence the need for collective saving.

*“We have these old men and women who cannot afford to pay on their own. They can join together in a group and invite their Member of County Assembly (ward representative) to conduct a harambee (public fundraising) for spare parts,”* said an opinion leader.

Although group savings was perceived as a good way of generating sufficient resources to purchase spare parts, there was some distrust about group efforts that involve money. Many respondents were wary of group savings and narrated previous bad experiences with group undertakings that involved money.

*“No! I have an experience with things like this being done in groups and they don’t succeed. Things don’t work in the correct way in groups because you can put money together and then you find the money is not there,”* said an opinion leader.

### Construction and evaluation of sustainability options

#### Sustainability options

The data from interviews with CAB members and opinion leaders were used to construct preliminary sustainability options in Table 1. Sustainability option A leans towards a more individual orientation. In this option, households own SMoTS and individually pay for spare parts and repairs. Option B is similar to the former with the distinction that SMoTS’ owners may form voluntary savings groups to finance parts and repairs. In options C and D, there was a governing body which owns SMoTS on behalf of households. But while in option C households form compulsory groups in which they save money that the governing body uses to stock the spares parts shop, in option D, a donor finances stocking the shop and households pay a monthly fee to the governing body to cover individual household’s spare parts and repair needs.

**Table 1:** Sustainability options A, B, C, and D and their features in terms of key components

	Options			
Key component	A (Individual household)	B (Household/ voluntary groups)	C (Governing body + compulsory groups)	D (Governing body + donor)
Governing body	No	No	Yes	Yes
Ownership	Individual	Individual	Governing body	Governing body
Repairs	Self/technician	Technician	Technician	Technician; paid by governing body
Shop in Mbita	Yes	Yes	Yes	No
Monthly fee to governing body	No	No	Yes; to fund shop stock)	Yes; to fund repairs/technicians
Saving in groups	No	Optional	Compulsory	Optional
Donor	No	No	No	Yes; to fund shop stock

#### Community members’ evaluation of sustainability options

The four sustainability options were presented to some community members to express their preferences during FGDs. The feedback, summarised in Table 2, suggests that respondents generally preferred sustainability option A followed by B. In general, respondents would like to own SMoTS and finance spare parts and repairs but that someone else procures and stocks affordable and accessible spare parts as well as have access to technicians. While option C contained collective elements that were preferred by the project management and some CAB members and opinion leaders, it gained little or no support in the community.

**Table 2:** Community members’ evaluation of sustainability options

	Options			
Response category	A (Individual household)	B (Household/voluntary group)	C (Governing body + compulsory groups)	D(Governing body + donor)
General views	The preferred option	May work for some	May work for villages and some beach women but with conditions	May work for villages and some beach women
Conditions			Sense of ownership and trust in governing body	
			Addressing the risk of misappropriation of contributed funds	
			Governing body composed of SMoTS owners	
Advantages/Conditions	Households accountable to themselves on sustainability	Group members will support the poor to maintain SMoTS		Donor provides funds for stock
Disadvantages	Difficulty recovering credit	Free riders	Distrust in governing body	Distrust in governing body
	Good for light but not for malaria elimination	Misappropriation of group resources	Bad previous experiences with group monetary contributions	Lack of trust among people on island
	Untrustworthy technicians	Lack of trust between members	Governing body could favour parts of the island	Failure to pay monthly fee
	Neglect/sale if high maintenance costs	Frequent migrations in town areas		
	Clarify SMoTS owners in beaches; landlord or tenant	Politics		
		Gossip		

#### Views and developments on a governing body and group formation

Besides yielding arguments similar to those during interviews, the discussions during the focus groups revolved around the axis on which the options presented were different: having a governing body or not, and the appropriateness of having compulsory or voluntary savings groups. In the discussions, the two often appeared interlinked as both were associated with collection of funds.

In concurrence with results from interviews, savings groups were seen as a useful component, especially for financially weaker persons:

*“It can work since group work is inclusive of the old who do not have enough knowledge about the system. From the group they will get assistance: those that lack enough money will get assistance from the group savings; group work is also very good since people share ideas; working in a group can enable them face credit as a group thus making it easier; and, group work opens ways for other developments.”*

However, many respondents narrated negative experiences with groups related to poor group cohesion, misappropriation of funds, and gossip. According to some, these problems were also linked to specific parts of the island:

*“There are areas in Rusinga where groups can succeed and areas where they cannot succeed, people might contribute money and when I need some repairs there is no money.”*

*“It might not work in town since people keep migrating in and out and also forming groups in town is not easy because people vary in opinion and bringing them together is also a very big problem.”*

These concerns were exacerbated by previous experiences when leaders failed to account for finances, including a long-standing stalemate over Kenya Shillings (KShs) 500 (5 United States Dollar (USD)) presumably contributed to a CBO kitty by individuals in anticipation of household “prequalification” for SMoTS. The project clarified at start-up that distribution of SMoTS was to be determined by the scientific design of the study.

*“First that word governing body has scared me...in most cases projects have failed on Rusinga because of such bodies. Some time back there was this idea; KShs 500 that was collected from community and we don’t know where the money is, they also have harsh conditions that if you can’t meet they will take the systems. Without saying too much I can’t support that model. No!”*

Respondents suggested mechanisms through which group problems could be ameliorated, such as joining or forming groups of their own choice and with people they trust:

*“They should identify their own membership and they should be people that live close to one another since if someone comes from far you might not know his or her character i.e. knowing one another better before coming together to form a group.”*

During a CAB meeting in June 2015, it emerged that an existing women’s table banking group (group savings and loaning) had incorporated saving money towards future maintenance of group members’ SMoTS. From savings made from kerosene purchases as a result of owning SMoTS, members contributed KShs 100 (1 USD) monthly into a SMoTS maintenance kitty. Membership consisted of people who knew and trusted each other, and the group had rules which included expelling non-compliant members. The 20-member group did not intend to grow larger to avoid reduced individual commitment.

Based on the outcomes of this study and the women’s group experience, the project adopted the group saving approach by seeking to encourage integration of savings for SMoTS into existing table banking groups and encouraging formation of similar groups across the island. The project engaged the champion for table banking to coordinate this activity. By December 2015, 86 savings groups had been sensitised and 48 were saving towards sustaining members’ SMoTS. Project monitoring of savings groups was on-going and will provide vital lessons going forward.

## Discussion

These findings indicate that residents leaned towards largely individualistic sustainability solutions. Selective collective solutions such as table banking groups were considered positively for mobilising financial resources for maintenance, but residents were hardly willing to contribute financially to a collective entity. Very few people saw a meaningful role for a collective governing body; people preferred to rely on individual household responsibility and private service delivery for repairs and stocking spare parts. As summarised in Table 3, there seemed to be linkages between preferences towards organising various components of SMoTS sustainability and social dilemma factors.

**Table 3:** Linkages between preferences towards organising sustainability components and social dilemma factors

	Sustainability components			
Social dilemma factors	Ownership	Repairs	Stocking and selling parts	Financing parts
Trust	Yes: Individual ownership due to low trust in collective ownership body.	Yes: Individual pay arrangements due to low trust in collective body and technicians.	Yes: Private business venture.	Yes: Individual approach due to concerns about unaccountable leaders and free riding group members.
Group identity	No	No	No	Yes: Individual approach and/or self-selected group memberships.
Leadership	Yes: Individual ownership due to concerns about unaccountable group leaders.	Yes: Individual arrangements due to concerns about unaccountable leaders.	Yes: Private business venture.	Yes: Individual approach due to concerns about unaccountable group leaders.
Group size	Yes: Individual ownership to enhance accountability.	Yes: Individual arrangements due to concerns about unaccountable leaders and free riding group members.	No	Yes: Individual approach and/or groups with limited members due to concerns about unaccountable leaders and free riding group members.
Possibility of effective sanctions	No	Yes: Individual pay-per arrangements to control technicians.	No	Yes: Individual approach and/or groups that sanction non-compliance.

An overriding concern was that people lacked trust in other community members, leaders and/or technicians who would be employed by a governing body. This distrust was based largely on past experiences with free-riding and unaccountable leaders, including an experience related to this project. The story of the CBO collecting money “on behalf” of the project demonstrates how efforts to foster sustainability of initiatives from outside may already be undermined before a project starts and develops a sound understanding of the context in which it operates. Based on this and other experiences, residents also had little confidence that a governing body or saving group could effectively impose sanctions to misappropriation of funds, poor leadership, defecting group members or technicians that might abuse a salaried position. The large size of the community, and the diversity on the island in terms of sub-communities, in- and out-migration, and residence patterns (towns versus rural areas) seemed to also complicate the achievement of collective solutions.

In interpreting these findings, it is relevant to note that respondents may have been thinking mainly about benefits of house electrification which accrued to individual households and were relatively cheaper to maintain when expressing preference towards an individual approach. Earlier project research revealed that residents were more excited about electrification benefits [3], raising the question about the overall perceived value of SMoTS and the recognition of the possible public good benefit therein. Early evaluation results and perceived effectiveness of an intervention improves prospects for project continuation [29, 30] and may especially be relevant for the malaria control aspect, whose impact had not been analysed when this study was carried out.

Although SMoTS proved effective in controlling malaria [1], that may not assure sustainability as the problems and issues outlined above remain. A review of the sustainability of community-based tsetse fly control programmes in Kenya revealed that although the approaches were associated with good reductions in tsetse fly populations and trypanosomosis prevalence during the first few years, there was failure to build new traps and maintain traps. In Lambwe Valley, Kenya, insufficient funds were available from the community to maintain the traps. In Busia, most traps were destroyed by either rats or floods or people had believed the project was for the researchers and abandoned maintenance after the research period [31]. SMoTS, installed in homesteads, may enhance a sense of ownership and responsibility. House electrification may also increase the desire by community members to sustain SMoTS.

This study was part of the project hence part of project learning. While the project and CAB had initially considered organizing sustainability through an island-wide body, insights from social science research led to changes. After learning that an island-wide body may not work well, the project initially planned to organise savings groups according to metaclusters before learning that this was also not feasible as residents preferred self-selected group memberships. Through adaptive learning in-built into the action research focus of this study, the project adopted the group savings approach by seeking to encourage integration of savings for SMoTS into existing table banking groups and encouraging formation of new groups. This led to the involvement of a community champion to coordinate the efforts. Community champions are pivotal in triggering and sustaining behavioural change [32, 33], including the organisation and formation of new groups with which people can identify [34].

The experience with the table banking groups also demonstrated that some residents in the community (notably women’s groups) had already developed more or less effective solutions to some of the problems of trust, identity and leadership that the project was struggling with in ensuring sustainability. It was important therefore that the project actively looked for these and tried to build on them.

## Conclusion

This study suggests that residents of Rusinga Island leaned towards largely individualistic and privately organised solutions to maintenance of SMoTS. This posed considerable challenges to organising the sustainability of this innovative malaria control strategy, which had features of a social dilemma. There seemed to be linkages between preferences towards organising various components of SMoTS sustainability and known hindrances to addressing social dilemmas such as lack of trust, large group size, weak group identity, and limitations in leadership and the impossibility of imposing effective sanctions. These conditions were not very conducive to realizing the initial sustainability options that were considered by the project.

The community, however, provided vital lessons on how to organise this strategy to improve success. This learning was possible because of the project’s action research approach that generated feedback about community views and experiences as part of a reflexive process of research and monitoring. In hindsight, the prospects for sustainability would have improved if planning and research for sustainability had started well before the launch of the project. This might simultaneously have prevented that a CBO could illegitimately collect money from residents in the name of the project before the project even started; an event which complicated later sustainability efforts and haunted the project from the start.

Improving prospects for sustainability require active attempts to improve trust in collective community undertakings. This may be done through showcasing successful group undertakings such as women’s groups’ table banking, allowing self-select group memberships, and developing linkages with existing programmes and organisations in the community.

## Abbreviations

CAB: Community advisory board
CBO: Community-Based Organisation
FGD: Focus group discussion
SMoTS: Solar-powered mosquito trapping system
